# The Gluten-Free Diet in the 3rd Millennium: Rules, Risks and Opportunities

**DOI:** 10.3390/diseases3030136

**Published:** 2015-07-13

**Authors:** Lori Welstead

**Affiliations:** Section of Gastroenterology, Hepatology and Nutrition, University of Chicago Medicine, Chicago, IL 60637, USA; E-Mail: lori.welstead2@uchospitals.edu; Tel.: +1-773-702-0019; Fax: +1-773-702-7782

**Keywords:** gluten-free, diet, celiac disease, nutrition, cross-contamination

## Abstract

The gluten-free diet has long been considered the standard treatment for celiac disease. However, a significant number of patients continue to experience persistent symptoms despite following a gluten-free diet. Inadvertent gluten ingestion, fermentable carbohydrates, cross-contamination, and social or financial burdens present obstacles to maintaining a gluten-free diet. Proper diet education and follow-up by an expert Registered Dietitian (RD) is essential to ensure adequate nutrition on the gluten-free diet. Patients may experience unintended weight gain or elevated cholesterol levels after initiating the gluten-free diet due to adequate absorption and healing of the intestines. This review deals with the evolving gluten-free diet, optimal recommendations while considering the overall health of patients, and multi-factorial aspects of the permanent lifestyle change.

## 1. Introduction

Celiac disease is a chronic, systemic, immune-mediated disorder triggered by exposure to gluten in genetically predisposed individuals. [1]. Over the years, overt gastrointestinal symptoms that almost invariably previously accompanied a diagnosis of celiac disease have become less frequent and obvious, posing additional challenges to clinicians. An Italian study found the clinical presentation drastically shifted over the 15 year interval of the study [2]. The majority, 66%, were found to have non-classical symptoms like bloating, osteoporosis and anemia. Only 34% had classical symptoms of diarrheaand malabsorption [2].

Medical treatment for celiac disease consists of life-long avoidance of gluten-containing foods. Gluten is used to describe specific amino acids in wheat (gliadin), rye (hordein), barley (secalin), and derivatives of these grains. Strict adherence to the gluten-free diet often minimizes bloating, diarrhea, weight loss, fatigue, and begins the healing process. Some patients feel improvements within days of starting the diet, although it may take up to a year for complete intestinal healing. This may be due to the degree of intestinal damage, or intermittent, unintended gluten ingestion.

## 2. History of Gluten and Wheat Production

Wheat gluten was first isolated in 1745 [3]. Since then, there have been constant advances in the production and usage of wheat gluten. Gluten, which means glue in Latin, provides a viscoelastic network [4] in food. These proteins produce the wide variety of textures, including crispy, crunchy, flaky and fluffy, in baked products.

Harvesting and consumption of cereals increased gradually over time, especially between World War I and World War II. In 1941, the Nutrition Society was formed in Britain to promote advances in nutrition and to arrange a rationing system for an increasing population. Its main objective was to increase global wheat production [5]. This shift correlates with an increase in the population of celiac disease: It was estimated that in 1950, 1 in 8000 in the United Kingdom had celiac disease [6]. Today, approximately 1% of the population in most Western countries has celiac disease [7].

Dicke initiated experiments of wheat-free diets in 1934–1936, and in 1950, published his thesis that found that gluten caused anorexia, increased bowel movements and steatorrhea. Dicke’s thesis provided the framework for the development of the gluten-free diet [8].

## 3. Nutritional Considerations

The duodenum is the uppermost section of the small intestine. It is lined with villi, small finger-like protrusions, which increase the surface area over which nutrients are absorbed. The villi themselves have microvilli on their tips, known as the brush border of the small intestine, which is responsible for breaking down proteins and a variety of sugars including lactose [9].

If an individual with celiac disease consumes gluten, the immune system responds by damaging the small intestine, and particularly the villi. At diagnosis of celiac disease, the villi may be partially shortened or completely flattened. Without villi, the ability to properly digest and absorb nutrients diminishes considerably.

Patients with celiac disease typically have malabsorption issues leading up to the time of diagnosis. Therefore, it is important to check for deficiencies upon confirmation of the diagnosis. There is a pressing need for international guidelines for vitamin and mineral supplementation in celiac disease. The most common nutrient deficiencies in patients with newly diagnosed celiac disease are iron, Vitamin B_12_, folate, calcium, Vitamin D, and zinc. The risk of nutrient deficiency is related to the degree of malabsorption, inflammation, or intestinal damage upon diagnosis [10]. If deficiencies exist, vitamin or mineral supplements are required to aid in proper nourishment and repletion. A multivitamin with minerals initiated at diagnosis can promote proper healing and compensate for the lack of fortification in gluten-free food products [11].

The gluten-free diet can be balanced and healthy with the inclusion of foods that are naturally gluten-free before processing. However, many patients are drawn to the convenience of processed gluten-free foods that are high in fat, sugar, and sodium without vitamin and mineral enrichment or fortification. Patients should be steered toward nutrient-dense foods, which are naturally good sources of these vitamins and minerals, to provide a balanced and adequate intake. These include fruits, vegetables, beans, nuts, seeds, fish, lean meat, poultry and dairy.

### 3.1. Iron

Iron-deficiency anemia has been detected in 46% of subclinical celiac disease cases, with a higher prevalence in adults than children [12]. Iron deficiency anemia in celiac disease is primarily due to malabsorption, as the duodenum, where villous atrophy is mostly located, is the main site of iron absorption. Both ferritin and CBC should be checked upon diagnosis [13]. Good sources of iron include beef, turkey, liver, egg yolks, sardines, and oysters. Treatment consists of a strict gluten-free diet and iron supplementation until iron stores normalize. This can take some time, typically shorter for children, but up to a year for hemoglobin and two years for iron stores to stabilize in adults [12].

### 3.2. Vitamin B_12_

Vitamin B_12_ deficiency is reported in untreated celiac disease patients at rates of 8% to 41%. It correlates with extensive disease, as most absorption takes place in the ileum [12]. The gluten-free diet, along with a multivitamin, is often sufficient for repletion of vitamin B_12_, although additional supplementation may be necessary. Dietary sources of vitamin B_12_ include shellfish, liver, dairy, beef, eggs. Vegans are at risk due to lack of B_12_ in the diet considering avoidance of all animal products. Elevated levels of serum methylmalonic acid (MMA) may enhance the diagnostic accuracy if vitamin B_12_ levels are in the lower range of normal or if there is an additional folate deficiency [14].

### 3.3. Folate

Studies have shown that 35%–49% of newly diagnosed celiac patients have folate deficiency [13]. Folate levels should be checked at diagnosis and annual follow-up visits. Unlike their gluten-containing counterparts, gluten-free foods are not fortified with folate. Women of childbearing age must be especially mindful of adequate folate levels and intake due to the importance of folate to the developing fetus.

### 3.4. Bone Health

The connection between bone health and celiac disease has been well documented [15]. One review suggests 75% of untreated adult patients with celiac disease suffer from a loss of bone mass [16]. Bone mineral density is related to inflammation from active disease as well as poor absorption. Calcium and vitamin D are absorbed in the duodenum. Clinicians should check 25-hydroxy-vitamin-D and in adults obtain a bone mineral density test at diagnosis, to determine baseline bone health and need for supplementation. The bone mineral density test should be repeated within a year if the patient is found to have osteopenia or osteoporosis [15]. Bone density increases over time on the gluten-free diet, and especially rapidly in children; as the risk is reduced with good dietary adherence and reduction in intestinal villous atrophy [17]. Ideally, between the diet and supplements, adult patients with celiac disease should have a calcium intake of at least 1000 mg per day [18]. Many patients experience lactose intolerance which leads to gas and bloating that may overlap symptoms of celiac disease. Lactose intolerance may occur if villi are damaged, as lactase enzymes are in the brush border at the tips of the villi; but often improves with intestinal healing. Patients who suffer from gas, bloating, and diarrhea at diagnosis should be advised to avoid lactose to reduce symptoms [9]. Often after several weeks to months on a gluten-free diet, lactose containing products can be reintroduced without overt symptoms of intolerance.

## 4. Rules of the Gluten-Free Diet

The medical treatment for celiac disease is a life-long avoidance of gluten-containing foods. G*luten* is found in wheat (gliadin), rye (hordein), barley (secalin), and derivatives of these grains. Oats are not included in this group, but must be regarded with caution, due to risks of cross-contamination. The first step in managing the gluten-free diet is to understand which foods contain wheat, rye, and barley so they can be eliminated from the diet, and intestinal healing can begin. Fresh foods, without any processing or additives, from fruit, vegetables, dairy products, fish, and meat, meat alternative food groups are all naturally gluten-free. The diet is complicated and confusing, with misinformation traveling over the Internet and bewildering patients. The gluten-free food market continues to grow: some estimates state that gluten-free sales will reach $15.6 billion in 2016 [19].

### 4.1. Oats

Oats are a significant source of vitamins, minerals, and heart healthy soluble fiber. Thompson and colleagues studied inherently gluten-free grains, and found gluten in 32% of samples not labelled gluten-free [20]. This prompted recommendations to only consume oats labelled as gluten-free, as the manufacturers follow a purity protocol to reduce the risk of cross contamination. The purity protocol involves frequent sampling of gluten-free oats (in the field, during shipment, processing and in storage facilities) to reduce the risk of cross-contamination [20].

### 4.2. Gluten Allowance in Gluten-Gree Goods

The FDA set a gluten limit of less than 20 parts per million (ppm) in foods that carry the label gluten-free [21]. This is similar to guidelines set by the Codex Alimentarius [22] and the European Union (EU) [23]. The EU guidelines consider 20 ppm to be gluten-free and <100 ppm gluten as “very low gluten” [23]. Since August 2013, a food labeled “gluten-free” that fails to meet the requirements of the regulation will be subject to regulatory action by the FDA.

According to a study by Catassi, 50 mg of gluten introduced daily induced villous damage over three months [24]. Researchers found a significant decrease in the villous height to crypt depth (vh/cd) ratio in the group taking 50 mg gluten daily. No significant change was found in the vh/cd ratio in the group taking 10 mg gluten daily for three months. This study provided evidence for avoidance of minute amounts of gluten, with the cut-off of 10 mg gluten daily [24,25]. One would need to eat more than a pound of food (500 g) tested at 20 ppm of gluten to achieve 10 mg gluten in a day.

### 4.3. Cross Contamination

Gluten exposure from cross-contamination may lead to ongoing disease activity, and is of concern. Considering risk of cross-contamination, it is recommended to avoid grains and flours from bulk bins, and to purchase flours and grains labeled gluten-free [11]. Condiment containers that allow use of spoons or other utensils may be at risk of cross-contamination in shared kitchens. Deli slicers, cutting boards, toasters, and colanders are risks of cross-contamination in shared kitchens.

A gluten-free diet has become easier to follow with the explosion of gluten-free products and increasing options available when eating out. A major concern is eating out; as the establishment may have best intentions in mind but there is often cross-contamination. Despite a gluten-free menu, many restaurants do not follow strict guidelines to avoid cross-contamination. If the wait staff of a restaurant does not communicate closely with the kitchen; this poses risks as the gluten-free order may not be high priority, or overlooked. Fortunately, chefs have become more aware of the gluten-free diet and concerns regarding cross-contamination over the past decade [26].

### 4.4. Education

Proper gluten-free diet education and counseling by an expert RD is necessary to aid in adherence to the gluten-free diet. After the initial visit, the patient should have access to a RD to answer questions as he or she learns to navigate the gluten-free diet. Such access would ideally include follow-up visits, phone calls, as well as analysis of food logs to ensure compliance and understanding of the diet. Not all insurance carriers cover visits with a RD. It is advisable for patients to check insurance coverage before the visit to avoid billing issues.

Misinformation online can lead to anxiety, confusion, and overwhelm the patient. The expert RD should review the diet, food logs, and lifestyle with a “fine tooth comb” to identify potential sources of gluten exposure. Important areas to focus on include cross-contamination in the home, frequency and locations of eating out, and products purchased that may be cross-contaminated. The RD can direct patients to reputable information sources. Ludvigsson has four steps to assess diet adherence: dietetic review, serum antibodies, clinical assessment of symptoms and follow-up biopsy [27].

### 4.5. Testing

The R5 sandwich ELISA is a reliable test and used to test most foods worldwide. This test is unable to evaluate gluten content in foods with hydrolyzed proteins or fermented products. An example is the issue arising with gluten-removed beers. These beers are barley based, and are treated with enzymes that break the proteins at the amino acid proline. The R5 sandwich ELISA requires proline to be interpreted correctly. Therefore, no safe or accurate test is available to deem a gluten-removed beer safe for consumption [28]. Hopefully the future will bring advances with testing. It is advised to only consume gluten-free beers and avoid gluten-removed beer until an appropriate test is developed to ensure safety.

Only a handful of studies have been done to assess gluten content in foods that are labelled gluten-free. In the past year, three studies had varying results. One study analyzing 275 U.S. gluten-free products found 1.1% containing ≥20ppm of gluten [29]. Another study found vastly different results with nearly 20% of foods labeled gluten-free contained ≥20ppm of gluten [30]. A study by Thompson found 5% of 158 gluten-free products with ≥20ppm of gluten [31]. A study of European foods analyzed from Italy, Spain, Germany, and Norway found 99.5% of the foods were under 20 ppm and 94% were under 5 ppm [32].

## 5. Risks Associated with Strict Adherence to a Gluten-Free Diet Mercury

A recent study found a four-fold increase in mercury blood levels in celiac patients following a gluten-free diet [33]. This study took into account amalgam fillings which contain mercury, as well as seafood intake; which did not correlate with blood and urine samples of mercury. Mercury is primarily absorbed in the duodenum. This outcome could be due to an altered response to mercury exposure, with a predisposition to accumulate it. Further studies are needed to clarify the concern of mercury in celiac disease and inspire guidelines for the surveillance of mercury in food.

### 5.1. Rice

Rice is a main staple in the diet of many with celiac disease. Consumption of plain rice and convenience foods processed with rice has skyrocketed. These include rice crackers, rice milk, rice flours, rice noodles, which further increases the average intake. However, most varieties of rice have been shown to contain high levels of inorganic arsenic, a carcinogen [34]. This may pose concerns due to large volumes of rice consumed by those following a gluten-free diet. Large doses of arsenic are life threatening, but small doses may increase risk of heart disease, diabetes, and cancer [34]. In 2013, the Food and Drug Administration (FDA) conducted an analysis of 1300 rice-based foods to determine levels of arsenic. Varying levels of arsenic were found in the foods tested. The FDA plans therefore to conduct risk assessments as the analysis did not determine long-term risks, and it should also be noted that no “safe” levels of arsenic have been established by the FDA [35]. This furthers the importance of a diet with varied grains, an important consideration that could simply be overlooked in those who do not see a RD at diagnosis.

### 5.2. Corn

Corn has long been a staple in the typical gluten-free diet. Similar to rice, corn is one of the predominant gluten-free options in convenience foods, including corn tortillas, chips, or other baked foods or snacks. Corn has been found to contain high levels of mycotoxins, specifically fumonisins [36]. In the initial study done in the Czech Republic in 1998, 88% of corn-based foods tested had high levels of mycotoxin fumonisins [37]. It was specifically high in extruded corn products, including polenta, but lower in corn flour or corn porridge [37]. A more recent study in Europe found 90% of gluten-free foods were contaminated with fumonisins, although the overall median of these values was below the European Union limit for adult consumption, which is 800 µg/kg [38]. Approximately 17.5% of the products contained fumonisins above the legal limit, with up to 3310 µg/kg [38]. The conditions corn sustains during the growth in fields, storage and processing allows for fumonisins to fester. This is yet another reason to support a varied diet, a restrictive diet could prove hazardous.

### 5.3. Microbiota

A study in Spain found a significant decrease in lactobacillus and bifidobacterium, and fecal short chain fatty acids in celiac patients on a gluten-free diet for 2 years [39]. There was a reduction in the diversity of healthy bacteria in 5 of 11 treated celiac patients, although 6 of 11 had similar microbiota compared to healthy controls [39]. Another recent study found a decrease in lactobacillus in pediatric celiac disease patients following a gluten-free diet [40]. Further studies are warranted to determine if probiotics or prebiotics may be of benefit to increase beneficial bacteria in celiac disease.

## 6. Weight Gain

Gluten is versatile, as it provides a variety of functions in foods. When gluten is removed, the product is denser. Sugar and fat are often added to provide a similar mouthfeel, which can result in higher calories. A recent study in Spain analyzed the nutritional content of gluten-free and gluten-containing products, which revealed significant differences. Gluten-free products contained more fat, predominantly saturated fat, more sodium, and less fiber and protein than gluten-containing counterparts [41]. Recent utilization of bean and nut flours, which aid in the texture, composition and nutritivevalueof gluten-free foods, has made some gluten-free products quite similar to their gluten-containing counterparts [42] and may aid in reducing the effect of weight gain on a gluten-free diet.

Shepherd *et al*. found those following a gluten-free diet had some nutritional inadequacies and excesses. Those following a gluten-free diet consumed inadequate fiber and folate, with higher fat and sugar content of gluten-free foods [43]. Further attention should be paid to the importance of fortification of gluten-free foods to mimic gluten-containing counterparts.

It has been reported that between 39%–44% of patients are overweight or obese at diagnosis [44,45]. This reflects the general population with respect to overweight and obesity [45]. Approximately 81% patients with celiac disease gain weight on a gluten-free diet [44]. 

Metabolic syndrome is a cluster of risk factors for cardiovascular disease and type 2 diabetes, and encompasses abdominal obesity, high blood pressure, dyslipidemia and impaired glucose regulation [46]. The prevalence of metabolic syndrome is estimated to be approximately 20%–25% of the world’s population [47]. A recent study found nearly 30% of patients with normal weight at diagnosis shifted into metabolic syndrome after only one year on the gluten-free diet [48]. The researchers suggest an in-depth nutrition assessment and follow-up with a RD to help prevent weight gain and shifts in metabolism. 

A study at Columbia University found that education and counseling with a RD improved all categories of body mass index (BMI) [49]. In this study, 54% of overweight celiac patients and 47% of obese celiac patients lost weight after initiating a gluten-free diet [49]. Additionally, 66% patients who were underweight at diagnosis gained weight after a consult with a RD experienced in the gluten-free diet [49]. Cheng notes this further emphasizes the importance and impact of RDs in gluten-free diet adherence and quality of life. Expert diet counseling should encompass multi-factorial aspects of patient’s health, including obesity, high cholesterol, diabetes, and food allergies or intolerances. In 2012, Green stated, the “risk of weight gain needs to be addressed by the gastroenterologist, the primary care physician, and the registered dietitian” [50].

In a study of more than 1000 patients over three years, 22% of patients that complied to the gluten-free diet gained weight, increasing their BMI >2 pts. At diagnosis, 20.5% were overweight and 11.5% obese [51]. Over three years on the gluten-free diet, 17% who started at normal weight crossed over into overweight or obesity. Fortunately, 19% who started out overweight shifted to normal weight [51]. 

### Heart Health

In a study at the Cleveland Clinic, there was a two-fold increase in coronary artery disease in celiac disease versus non-celiac disease patients [52]. A study in Sweden found cardiovascular disease risk increased 60% after diagnosis of celiac disease [53]. This is of interest because systemic inflammation, as well as a healed small bowel, may result in elevated cholesterol levels.

## 7. Persistent Symptoms Despite a Gluten-Free Diet

A study of over 1200 celiac patients found 23% considered as non-responsive celiac disease (NRCD) [54]. This study provided diet education to non-responders by a RD on a strict, gluten-free diet termed the “gluten-contamination elimination diet.” The diet emphasized avoidance of processed foods and minimized risk of cross contamination. After initiation of a strict gluten-contamination elimination diet, there were significant changes. Eleven of 14 subjects experienced resolution of symptoms, and were able to return to a normal gluten-free diet without issues. Five of six patients with Marsh 3 lesions on biopsy had resolution of symptoms and were no longer considered to have refractory celiac disease (RCD). Another recent study showed that in 90% of patients considered NRCD; the problem was due to continued gluten ingestion [55]. 

If a strict gluten-free diet does not resolve symptoms, it is important to consider a consultation with a RD to rule out obvious or inadvertent sources of gluten. If antibody levels are improving or normal but symptoms persist, consider other reasons for persistent symptoms. These considerations may include fermentable food molecules, small intestinal bacterial overgrowth, lactose or fructose intolerance, microscopic or collagenous colitis, irritable bowel syndrome (IBS), inflammatory bowel disease (Crohn’s disease or ulcerative colitis). There is often an overlap of IBS with celiac disease. A meta-analysis of over 3000 patients found that 40% of those with celiac disease also experienced IBS [56]. 

Paarlahti *et al.* found that 25% of patients suffered from persistent symptoms despite following a strict gluten-free diet. Approximately 78% suffered from depression [57]. Anxiety, stress and depression can affect the ability to follow a strict gluten-free diet. Verdu suggests an overlapping pathophysiology of celiac disease, IBS, gluten sensitivity and other food intolerances [58].

Many patients experience gas, bloat, reflux, constipation and loose stools, despite initiation of the gluten-free diet. Some may have a heightened awareness of symptoms after initiating a gluten-free diet. Often, gluten-free sweets, granola bars, or other foods have added sugars or sugar alcohols to provide an acceptable product. Some are high in fermentable carbohydrates, which if malabsorbed could mimic symptoms of celiac disease. Fermentable dietary molecules (FODMAPs) are, “widespread in the diet and comprise monosaccharide (fructose), disaccharide (lactose), oligosaccharides (fructans and galactans), and polyols. Their ingestion increases delivery of readily fermentable substrate and water to the distal small intestine and proximal colon, which are likely to induce luminal distension and induction of functional gut symptoms” [59].

## 8. Opportunities

In the area of gluten-free oats, there may be opportunity for oat cultivators to develop hybrid oats that are less likely to exacerbate the inflammatory response in celiac disease. A study out of Italy assessed duodenal biopsies to determine which cultivars of oats caused an inflammatory response similar to wheat. Gliadin-induced transglutaminase-2 (TG2) was measured on the epithelial lining to determine reactions. Three types of cultivars were reviewed: Nave, Potenza and Irina [60]. Nave cultivars were the only type of oat to significantly cause inflammatory response to TG2. This may help many to vary the gluten-free diet, especially those avoiding gluten-free oats due to overt symptoms.

In the future, RDs can instruct celiac patients to enjoy and follow an adequate, balanced gluten-free diet [61]. Inclusion of education regarding weight management, heart healthy diet, low FODMAP, and consideration of IBS in celiac disease management is appropriate to avoid concomitant diseases or concerns in the future. Food companies and restaurants certainly have a role to play in the future to ensure that foods are properly tested to provide safe gluten-free products to consumers.

Education about the gluten-free diet and its potential pitfalls are essential for successful adherence. Often, patients are not satisfied with their physician or RD visit when diagnosed. Unfortunately not enough RDs are specialized in the education of a strict gluten-free diet. This is a gap in the medical system, as coping strategies are not consistently included in medical or nutritional plans [62].

In addition, the way patients and their families perceive the diagnosis of celiac disease affects compliance with diet. Anxiety, depression and fatigue are often linked with celiac disease before and after diagnosis [27]. Patients should have access to support groups; whether online or a local group to provide additional support. Adherence to the gluten-free diet with adequate symptom control improved health related quality of life [63]. Lifelong commitment of a strict gluten-free diet affects emotional and cognitive aspects, and interpersonal relationships. A recent study found a significant correlation between non-compliance and depression with a decrease in quality of life [64]. Barriers of compliance could minimize the therapeutic effect of the gluten-free diet. This could be likened to Type 2 Diabetes; as patients may not follow recommendations, despite education, including risks and complications. Not all are ready or willing to go gluten-free, especially those that may not have overt symptoms.

Patients on a gluten-free diet perceive limitations on their social life including travel, eating out, and navigating food at parties. A study of approximately 1700 patients found celiac disease significantly affected quality of life, including economic, social and physiological aspects [65]. An economic burden falls on patients and families, as children and adolescents are not able to consume foods often provided at school and require additional steps to ensure gluten-free status of institutional food. Guandalini noted that “Effective communication between the physician and the patient is essential in minimizing the disease burden of celiac disease, particularly in young patients” [66].

Motives to maintain a strict gluten-free diet vary significantly from patient to patient. A recent study found a significant correlation of following the gluten-free diet in those diagnosed with celiac disease with improvement of general physical symptoms and the feeling of hitting “rock bottom” with symptoms [61]. Patients aware of long-term health consequences maintained the diet better. “Fear was the motivation to start and stick with the diet.” It is not simple to understand the myriad of factors surrounding motivation [61]. This provides opportunity for clinicians, as well as basis for education at diagnosis to understand the disease and concerns of not remaining gluten-free.

## 9. Conclusions

The gluten-free diet is the only treatment for celiac disease. There are many factors to ensure adherence to the diet. Proper education by an expert registered dietitian, support, and follow-up are necessary components to aid in permanent lifestyle change. Education on balanced and varied gluten-free diets is pertinent, as many fall into a holding pattern with gluten-free choices. The safety and consistency of gluten-free products has advanced, but more progress needs to be made to ensure validity of testing. The gluten-free diet has evolved, providing future opportunities for clinicians, consumers, restaurants and the food industry.
